# Association of opioid use with survival in patients with cancer treated with immune checkpoint inhibitors: it is time for evidence-based behaviors

**DOI:** 10.1093/oncolo/oyae081

**Published:** 2024-04-30

**Authors:** Raffaele Giusti, Giampiero Porzio, Marco Maltoni, Marco Filetti, Arturo Cuomo, Elena Bandieri, Dario Trapani, Eduardo Bruera

**Affiliations:** Medical Oncology Unit, Azienda Ospedaliero Universitaria Sant’Andrea, Rome, Italy; Tuscany Tumor Association, Florence, Italy; Department of Medical and Surgical Sciences, University of Bologna, Bologna, Italy; Phase 1 Unit, Fondazione Policlinico Universitario A. Gemelli IRCCS, Rome, Italy; Department of Anesthesia and Critical Care, Istituto Nazionale Tumori-IRCCS, Fondazione Pascale, Naples, Italy; Oncology and Palliative Care Units, Civil Hospital Carpi, USL, Carpi, Italy; European Institute of Oncology, IRCCS, Milan, Italy; Department of Palliative, Rehabilitation, and Integrative Medicine, University of Texas MD Anderson Cancer Center, Houston, TX, United States

**Keywords:** immune checkpoint inhibitors, cancer pain, opioids

## Abstract

Cancer is a leading cause of morbidity and mortality worldwide, with pain experienced by most patients undergoing cancer treatment. Opioids are the recommended treatment for cancer pain management, but recent studies suggest a negative association between opioid use and survival rates among patients undergoing immunotherapy. However, conclusions cannot be drawn regarding causality from these observational data. Immunotherapy, which boosts the body’s immune system to fight cancer cells, has emerged as a promising treatment option for all types of cancer. Immune checkpoint inhibitors (ICIs) can activate the anticancer function of exhausted T cells and have shown remarkable survival benefits in patients with multiple malignancies. However, a recent systematic review and meta-analysis suggested that the use of opioids during ICI treatment has an adverse effect on patient prognosis, while the use of NSAIDs is not significantly associated with the prognosis in patients treated with ICIs. These reviews have major limitations due to the retrospective nature of the studies and the multiple factors that can influence the phenomenon. Therefore, caution is required when interpreting results from retrospective data on drug interactions. The findings of this study are alarming and potentially harmful to patients with cancer suffering from pain or other symptoms requiring opioid drugs.

## Teaser

A recent review suggested that the use of opioids during immune checkpoint inhibitor (ICI) treatment has an adverse effect on patient prognosis. This article urges caution when interpreting the results.

**Implications for Practice**

- Cancer pain management: effective pain management is crucial for alleviating suffering and maintaining an acceptable quality of life for patients with cancer. Opioids are recommended as the primary treatment for cancer-related pain, but the nuanced approach to dosage and the potential negative association with survival rates call for careful consideration in their administration.
- Impact of immunotherapy: the emergence of immunotherapy as a promising treatment for various cancers has reshaped cancer treatment strategies. However, conflicting results regarding the immune effects of opioids in patients with cancer and the potential adverse effect of opioids on patient prognosis during immunotherapy treatment raise concerns and necessitate a cautious approach.
- Need for comprehensive research: the existing literature underscores the need for comprehensive research, including randomized controlled trials, to elucidate the complex relationship between opioids and immunotherapy. Patient population, intervention types, and outcome measures must be carefully considered to draw meaningful conclusions.
- Evidence-based practices: while opioids remain essential for cancer pain management, efforts to minimize their use through personalized approaches, deprescribing, and the exploration of alternative adjuvant drugs are crucial. Prescriber education, guideline implementation, and careful evaluation of patients’ pain management needs are essential for optimal cancer care.

Cancer, a leading cause of global morbidity and mortality, accounted for 18.1 million new cases and 9.6 million deaths in 2018. Pain affects a significant proportion of patients with cancer undergoing treatment, with 55% experiencing it during anticancer therapy and 66% in advanced, metastatic, or terminal stages.^1^ Effective cancer pain management is crucial for alleviating suffering and maintaining an acceptable quality of life. The American Society of Clinical Oncology recommends opioids as the primary treatment for cancer-related pain, aligning with the World Health Organization’s recognition of opioids as essential drugs for managing moderate to severe cancer pain.^2,3^

The administration of opioids varies based on the context and indications for cancer pain. Short-term use may occur perioperatively, while long-term use is common in patients with ongoing cancer pain. However, there is a nuanced approach to dosage, as lower doses are often suggested for shortness of breath compared to what some patients need for overall cancer pain management.

Recent studies have reported a negative association between opioid use and survival rates, but causality remains unclear due to potential confounding factors.^4^ Tumor-related factors, patient pain severity, and other variables make it challenging to draw definitive conclusions from observational data. Moreover, the impact of morphine, a prototypical opioid, may differ from other opioids, highlighting the need for comprehensive studies encompassing various drugs and treatments.

In recent years, immunotherapy has emerged as a promising treatment for various cancers, leading to a shift away from traditional chemotherapy. The approval of multiple immunotherapy agents has reshaped cancer treatment strategies, with targeted therapies gaining prominence. The use of immune checkpoint inhibitors (ICIs) has shown remarkable benefits in activating anticancer functions, particularly in patients with multiple malignancies.

Several human studies examining the immune effects of morphine in patients with cancer yielded conflicting results, with no clear clinical impact assessed. The introduction of ICIs has seen continuous growth, both in adjuvant/neoadjuvant settings and palliative care, complicating the assessment of opioid effects on overall survival. A systematic review indicated an adverse effect of opioids on patient prognosis during ICIs treatment, raising concerns about the potential limitations and biases in retrospective studies.^5^

Several limitations in the existing literature underscore the challenges in interpreting the association between opioids and immunotherapy outcomes. Retrospective studies, heterogeneity in opioid usage across hospitals, lack of detailed reporting on opioid dosage, and the absence of crucial prognostic factors contribute to methodological flaws. The impact of opioid usage on various cancer types further complicates the interpretation, emphasizing the need for cautious analysis (Table 1).

**Table 1.** Characteristics of negative studies on overall survival and opioid use.

Reference	Year	Study	Patient enrolled	Opioids	Other medications	Cancer	ICI	Analysis
Bironzo^6^	2019	Retrospective multicentric observational	64	NOS	No	NSCLC	NOS	M
Iglesias-Santamaria^7^	2020	Retrospective multicentric observational	102	NOS	Yes	All	All	U
Cortellini^8^	2020	Retrospective multicentric observational	1012	NOS	Yes	All	All	M
Taniguchi^9^	2020	Retrospective monocentric observational	296	All	No	NSCLC	Nivolumab	U
Botticelli^10^	2021	Retrospective monocentric observational	193	All	Yes	All	All	U
Gaucher^11^	2021	Retrospective monocentric observational	372	NOS	Yes	All	All	M
Kostine^12^	2021	Retrospective monocentric observational	635	Morphine	Yes	All	All	U
Miura^13^	2021	Retrospective monocentric observational	300	NOS	Yes	All	All	M
Verschueren^14^	2021	Retrospective multicentric observational	442	NOS	Yes	NSCLC	All	M
Young^15^	2024	Retrospective monocentric observational	209	All	No	NSCLC	All	M

Abbreviations: NSCLC, non–small cell lung cancer; ICIs, immune checkpoint inhibitors; NOS, not otherwise specified; M, multivariate analysis; U, univariate analysis.

Despite evidence suggesting a potential negative impact of opioids on immunotherapy outcomes, caution is warranted. Generalizing findings without considering opioid types, cancer types, patient performance status, and life expectancy at treatment initiation may lead to misguided clinical practices.

The integration of immunotherapy near the end of life may create a mismatch between innovation access and palliative care, necessitating careful symptom management.

Existing evidence underscores the need for comprehensive research, including randomized controlled trials (RCTs), to elucidate the complex relationship between opioids and immunotherapy.

Investigating the impact of opioids on patients with cancer undergoing immunotherapy through RCTs raises ethical concerns. These trials may deviate from established pain management guidelines and introduce bias due to the complex medical backgrounds and medication regimens common in this patient population. Additionally, RCT recruitment may favor patients with less severe pain, potentially resulting in an unrepresentative sample.

Alternatively, a cohort study design presents a promising option. Patients starting immunotherapy would be longitudinally followed, enabling researchers to assess the influence of various factors, including symptoms management and opioid use, on survival outcomes. By circumventing the ethical dilemmas associated with RCTs, this methodology offers a more comprehensive understanding of the relationship between opioids, immunotherapy, and survival in patients with cancer.

Patient population, intervention types, and outcome measures must be considered to draw meaningful conclusions. While opioids remain essential for cancer pain management, efforts to minimize their use through personalized approaches, deprescribing, and the exploration of alternative adjuvant drugs are crucial. Prescriber education, guideline implementation, and careful evaluation of patients’ pain management needs are essential for optimal cancer care.

The emerging evidence indicating a potential impact on immunotherapy outcomes necessitates a cautious approach. A statistical association does not imply causation, and the use of opioids in conjunction with immunotherapy should not be dismissed outright. Evidence-based practices, guided by rigorous research and a nuanced understanding of patient needs, are essential for optimizing cancer care.

Continued investigation, incorporating dedicated study designs, is imperative for elucidating the intricate dynamics of opioid utilization within the framework of immunotherapy, thereby optimizing outcomes for patients with cancer.

## Data Availability

The data underlying this article will be shared on reasonable request to the corresponding author.
